# Training and Assessing Teamwork in Interprofessional Virtual Reality–Based Simulation Using the TeamSTEPPS Framework: Protocol for Randomized Pre-Post Intervention Study

**DOI:** 10.2196/68705

**Published:** 2025-05-27

**Authors:** Marie Lehmann, Jan Mikulasch, Horst Poimann, Joy Backhaus, Sarah König, Tobias Mühling

**Affiliations:** 1 Institute of Medical Teaching and Medical Education Research University Hospital Würzburg Würzburg Germany; 2 Department of Internal Medicine I Intensive Care Unit University Hospital Würzburg Würzburg Germany; 3 TeamSTEPPS Committee for German-Speaking Countries Würzburg Germany

**Keywords:** interprofessional teamwork, medical education, TeamSTEPPS, Team Performance Observation Tool, team training, Teamwork Perceptions Questionnaire, virtual reality

## Abstract

**Background:**

Interprofessional teamwork is essential for patient outcomes in emergency medicine; yet, effective training in this area is scarce. Virtual reality (VR) provides a promising, resource-efficient solution for simulating emergency scenarios and facilitating interprofessional collaboration. While VR-based training has shown benefits for medical skill and knowledge acquisition, assessing teamwork within such environments remains a challenge due to the lack of validated measurement tools. Existing teamwork assessment instruments, developed for physical simulations, may not fully apply to VR due to differences in communication modalities, interaction mechanics, and observer perspectives.

**Objective:**

This study aims to adapt and validate the TeamSTEPPS framework to assess teamwork in VR-based training. Subsequently, these adapted instruments will enable the investigation of whether interprofessional teamwork can be successfully trained in VR scenarios.

**Methods:**

Prior to the study, measurement instruments for subjective (Teamwork Perceptions Questionnaire) and objective teamwork quality (Team Performance Observation Tool, TPOT) will be adapted and validated for use in VR scenarios. Validation of the adapted version of the Team Performance Observation Tool includes expert consensus via a modified Delphi method as well as validity and reliability testing using recorded VR teamwork sessions. The study itself is designed as a prospective pre-post study with a planned enrollment of 65 nursing and 65 medical students working in randomly assigned interprofessional teams. On 3 timepoints (day 1, day 8, and day 15), participants engage in a VR scenario simulating 1 out of 3 different emergency medical conditions (esophageal variceal bleeding, exacerbated chronic obstructive pulmonary disease, and atrial fibrillation due to urinary tract infection). As an intervention, a structured training video on successful teamwork according to the TeamSTEPPS concept is shown on day 8 immediately before the second VR scenario. Teamwork is assessed objectively with the adapted version of the Team Performance Observation Tool and subjectively with the adapted Teamwork Perceptions Questionnaire. Medical performance will be recorded automatically by the VR software based on the medical measures conducted by the team.

**Results:**

As of May 2024, a total of 28 interprofessional teams have been enrolled. Data analysis will begin in late 2025.

**Conclusions:**

This study addresses the challenge of adapting teamwork assessment tools to VR environments and may provide insights into the potential of VR-based training for improving interprofessional collaboration in medical education. Future research could include a control group to measure the effects of team training more rigorously or use more enhanced technologies (eg, natural language processing) to capture the full range of teamwork behavior.

**International Registered Report Identifier (IRRID):**

DERR1-10.2196/68705

## Introduction

Interprofessional teamwork plays a crucial role in affecting patient outcomes in emergency medical contexts [1,2]. However, interprofessional team training is uncommon both in undergraduate medical education and in professional practice [3,4]. This is particularly due to the high personnel and resource requirements for effective team training [5,6]. Virtual reality (VR)–based training environments are playing an increasing role in emergency medicine, facilitating the widespread and effective learning of medical content, and may also enable interprofessional team collaboration training in the future [7]. The improvement in medical performance through such training is measured using various parameters such as checklists or “time to action” [8-11]. Some VR programs already allow for automated result recording [10], which could support examiners in complex practical examination settings in the future.

In contrast to capturing medical knowledge or performance, there are currently no validated tools for measuring (interprofessional) team collaboration in VR-based environments. For conventional physical simulation–based training, numerous tools exist to objectively capture various aspects of teamwork [12,13]. These are often based on Crew Resource Management or similar principles. However, these evaluation criteria cannot be fully transferred to VR scenarios for several reasons: Currently, team-capable VR training environments vary significantly in their level of functionality. For instance, considerable differences exist in how verbal interaction with virtual patients is represented (either through menu-based options or speech recognition [14]) and how haptic measures are depicted (solely via VR controllers [15,16] or additional haptic devices [8]). The ways teamwork is represented in VR environments (verbal communication, handing over objects, as well as briefing and debriefing) also may differ and nonverbal communication skills such as facial expressions and nuanced gestures are largely missing among current VR-based avatars. Finally, it is crucial to provide evaluators with a sufficient view of the actions and verbal communication in the VR scenario—whether as participants wearing a VR headset or through visualizing the participants’ perspectives on a computer screen. These challenges may explain why the quality of teamwork in VR-based environments has barely been measured so far. While technical hurdles are increasingly being overcome through advances in hardware and software, assessment tools for team collaboration in virtual settings still need to be adapted and validated.

A well-validated framework that includes educational content and both subjective and objective tools for assessing teamwork in physical simulations is the TeamSTEPPS framework [17]. In its current version (3.0), it covers 5 dimensions: team structure, communication, leadership, situation monitoring, and mutual support, and provides both subjective and objective evaluation metrics. The objective metric, the Team Performance Observation Tool (TPOT), has been significantly enhanced in terms of test-retest and interrater reliability through the inclusion of behavioral anchors compared to the original version [18]. In a recently published comparison of the most promising team performance assessment tools for evaluating longitudinal training, the TPOT was rated the most comprehensive tool and showed the second-best interrater reliability [19]. The associated questionnaire for subjective team member perceptions, the Teamwork Perceptions Questionnaire (T-TPQ), is also well-researched and validated [20]. While other assessment tools may outperform in specific criteria [12], the TeamSTEPPS concept is well suited for studies assessing comprehensive learning outcomes in simulations due to its alignment between teaching and assessment tools.

This study aims to adapt and validate objective and subjective assessment tools for the facets of teamwork that meet the requirements of VR-based environments, using the TeamSTEPPS measurement instruments. In the planned prospective study, these assessment tools will be used to measure the objective quality and the subjective perceptions of teamwork longitudinally (before, immediately after, and 1 week after completing a VR-based team training). In addition, the medical performance of the teams will be assessed based on the medical actions taken. The hypotheses derived from this are as follows:

- H1: A VR-adapted TPOT (vTPOT) supplemented with behavioral anchors demonstrates good item characteristics (concurrent validity, interrater reliability, and internal consistency) for the various facets of teamwork compared to the original version.
- H2: VR-based team training leads to a sustainable objective improvement in all facets of teamwork performance in interprofessional teams (as measured with the vTPOT).
- H3: VR-based team training leads to sustainable subjective improvement in all facets of teamwork perceptions in interprofessional teams (as measured with an adapted T-TPQ).
- H4: The increasing quality of teamwork is associated with an improvement in treatment quality, represented by the automatically recorded percentage of correctly performed medical actions.

## Methods

### Apparatus

**Figure 1:**
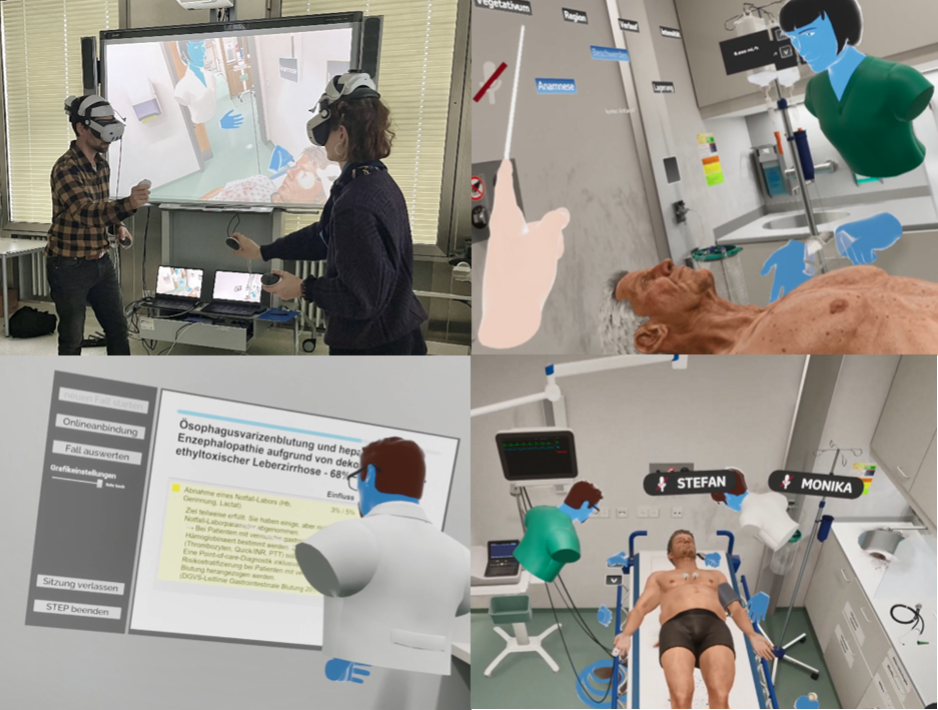
Top left: participants with the virtual reality head-mounted display and image transmission to a monitor (background) for the study staff. Top right: interaction of stylized avatars in the first-person perspective. Bottom left: final evaluation after the simulation with an assessment of medical performance. Bottom right: overview showing stylized avatars and a virtual patient.

STEP-VR (version 1.0; ThreeDee GmbH, Munich, Germany) will be used as the VR simulation of complex emergencies, which represents a joint development between a 3D visualization company and the University Hospital of Würzburg. The version includes multiuser functionality, allowing the selection of an avatar with typical nursing or medical attire and multiple stylized faces (Figure 1).

The VR hardware setup for this study includes 2 OMEN by HP 17-ck0075ng Laptops (chipset: Intel Core i7-11800H; graphics adapter: NVIDIA GeForce RTX 3070 Laptop GPU [8 GB GDDR6 dedicated]) and 2 Oculus Quest III VR head-mounted displays (HMDs). The equipment enables STEP-VR to run at a constant framerate of over 60 frames per second on “high quality” display settings of the HMD.

### Adaptation and Validation of the Measurement Instruments for Teamwork Quality

#### Adaptation and Validation of the TPOT for VR-Based Scenarios

The original version of the TPOT includes 25 items to assess 5 facets of teamwork: team structure, communication, leadership, situational awareness, and mutual support. Compared to traditional, in-person team training, the novel context of VR-based interactions, as well as the small team size of 2 people, make adjustments necessary. To ensure content validity, respective changes will be proposed by 4 experts—a certified TeamSTEPPS expert (HP), a specialist in internal medicine and medical education (TM), an intensive care nurse with additional specialization in medical education (JM), and the study coordinator (ML, a skills lab tutor with special training and 2 years of experience in undergraduate medical education)—independently and implemented after thorough joint discussion. In the next step, a modified Delphi method will be applied. Experienced doctors or nurses with either additional qualifications in medical education, specialization in emergency medicine or emergency nursing, or extensive experience in conducting team training form the expert panel. These experts will be asked to rate the appropriateness and applicability of items within the novel context of VR-based interactions [21].

To investigate the concurrent validity between the original TPOT and vTPOT, subsequently, 6 video recordings of teamwork in virtual reality scenarios will be evaluated by multiple raters using both instruments, and the correlation will be calculated overall and for each facet of teamwork. The interrater reliability for the vTPOT, as well as the internal consistency, will also be calculated from this analysis.

#### Adaptation and Validation of the T-TPQ for VR-Based Scenarios

The 35-item self-report tool T-TPQ developed by TeamSTEPPS, uses 5-point Likert scales to measure staff perceptions of Team Structure, Team Leadership, Situation Monitoring, Mutual Support, and Communication. These 5 facets of teamwork correspond to those described in the above-mentioned TPOT. The 5-point Likert scale ranges from 5=“strongly agree” to 1=“strongly disagree.” All items of the T-TPQ were translated into German.

For the identification of inappropriate items of the T-TPQ, quantitative and qualitative methods will be applied. In the first step, as part of a pilot study, all participants initially answered the 35 items of the original T-TPQ. Following this, internal consistency is calculated and items that strongly deviate from the responses to other items will be identified.

In the second step, an additional response option, “not applicable,” will be integrated into the T-TPQ for all items. Also, within the pilot study, participants will be asked to explain why they had rated an item as “not applicable.” Exclusion of inappropriate items will be made based on these analyses.

### Study Design

A prospective preintervention-postintervention study will be conducted between May 2024 and June 2025. Teams consisting of 1 nursing and 1 medical student will complete 3 different interprofessional VR-based scenarios on 3 dates. These teams will be newly randomized for each session.

Three scenarios, each lasting about 30 minutes, will be completed by all participants in random order on different dates: (1) esophageal variceal bleeding due to ethyl-toxic liver cirrhosis, (2) exacerbated chronic obstructive pulmonary disease, and (3) tachycardic atrial fibrillation due to complicated urinary tract infection. The contents of the scenarios and indicated medical actions are listed in Table S1 in Multimedia Appendix 1 and were already described in detail elsewhere [16,22]. Participants can communicate via voice-over IP and interact with each other through avatars during the scenarios (eg, handing over equipment or demonstrating findings on a screen). The type and order of medical actions and teamwork elements are not predetermined and can be independently structured by the team members (eg, briefing and mid-discussion). After the scenario, a detailed guideline-based evaluation of the medical actions and an overview of the physiological course of the disease can serve as the basis for debriefing.

**Figure 2:**
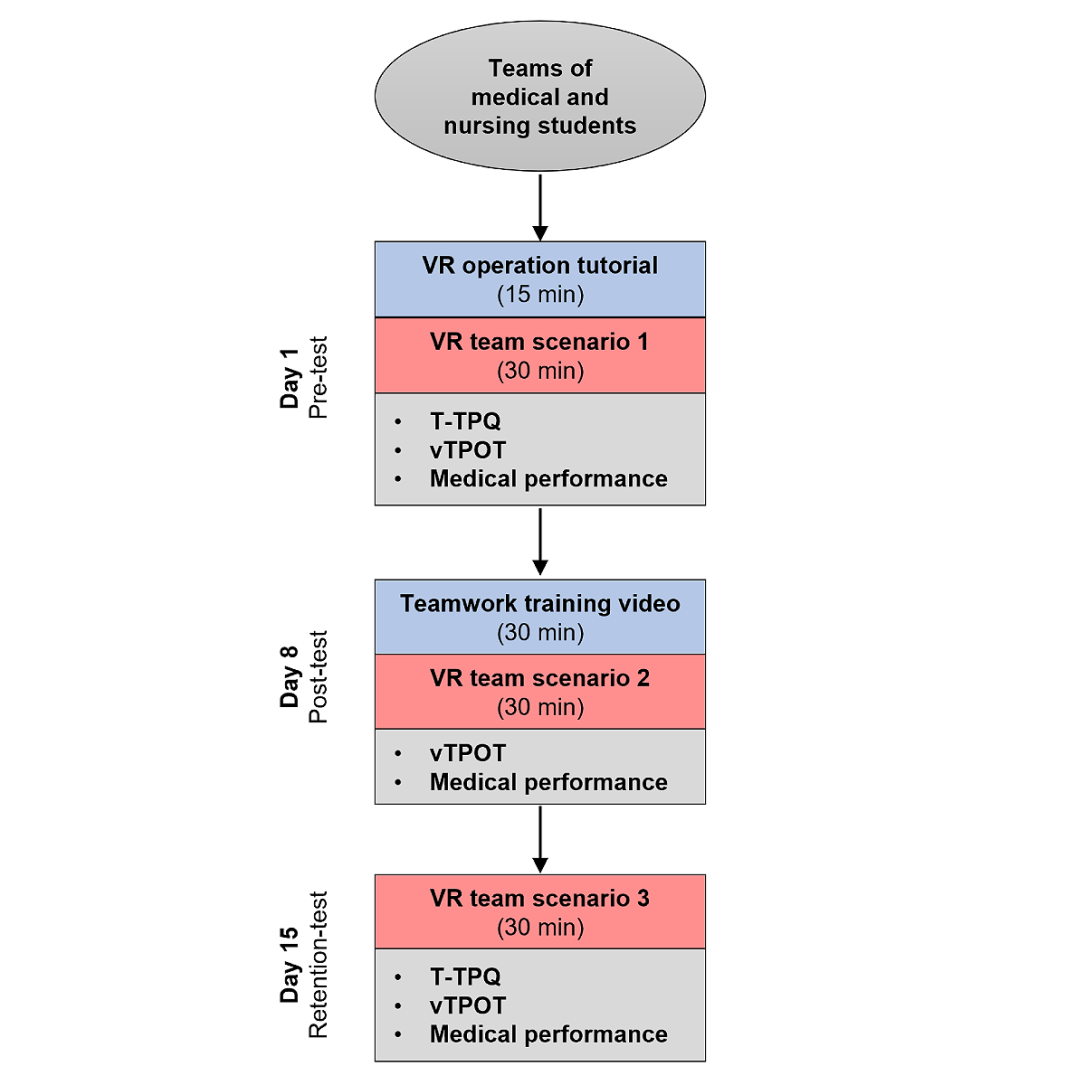
Overview of the data collection process. vTPOT: Team performance observation tool adapted for virtual reality scenarios. T-TPQ: TeamSTEPPS T-TPQ; VR: virtual reality.

On the first date (day 1), informed consent will be obtained. A tutorial will follow to familiarize participants with VR hardware and software. The first scenario will be completed without specific prior team training (pre-test). On the second date (day 8), a training video on successful teamwork including practical recommendations will be shown (approximately 30 min). The content of the training video is based on the official TeamSTEPPS guidelines [17] and has been adapted by the authors of the study to fit the specific context (limited time and VR setting). In addition, video examples were shown to demonstrate how certain team interactions (particularly for performing “Check-Backs,” “Huddles,” giving feedback, and applying the “Two-Challenge Rule”) can be implemented in VR. Then, the second scenario will be completed (post test). A total of 7 days later (day 15), without additional training, the third and final scenario will be completed (retention test). Figure 2 provides an overview of the study design and data collection.

### Participants

#### Overview

All regularly enrolled nursing students in their second-third year of training and all medical students in their 8th-10th semester at the University Hospital of Würzburg are eligible to participate. Participants of the study will provide written informed consent after being detailed on the study conditions. Inclusion criteria and exclusion criteria are listed in Textbox 1.

**Eligibility criteria.**

**Inclusion criteria**

- Regularly enrolled nursing students in their second or third year of training or medical students in their eighth to tenth semester
- Signed informed consent provided
- 18 years of age

**Exclusion criteria**

- Known epilepsy
- Known severe motion sickness

#### Sample Size Calculation

Since the team tandems are reassembled in a new, randomized form at each session a sample size estimation for 3 repeated measures timepoints could not be computed. Instead, we had to assume 3 independent groups. A between-groups ANOVA with an α level of 5%, a power of 80%, and assuming a large effect size, indicated that approximately 65 team tandems would be required, resulting in a total of 130 participants.

### Measurement Instruments

#### Measures of Teamwork Quality

Both measures of teamwork quality are based on the TeamSTEPPS framework and include items related to the 5 facets of team structure, communication, leadership, situation monitoring, and mutual support. Details on the adaptation and validation process are provided in the corresponding section.

Objective teamwork will be measured through first-person video recordings of the VR sessions, which will be analyzed using the adapted version of the TPOT (vTPOT). This tool will evaluate observable behaviors and team interactions based on predefined teamwork criteria.

Subjective teamwork will be assessed using the adapted version of the T-TPQ completed by the participants after the first and third VR scenarios, focusing on their perceptions of teamwork quality.

#### Automatically Recorded Medical Performance Score

After each scenario, both team members can view and discuss the final evaluation together. All medical tasks specific to each scenario are assessed and it is recorded whether they were fully completed, partially completed, or not completed at all. The checklists for each scenario had been previously established by the authors based on guidelines from professional societies [23-25]. It was already ensured as part of a pilot study, that the automated final evaluation—in comparison with a manual checklist—accurately recorded all measures [10]. Importantly, when recording the tasks, the VR software does not differ whether they were carried out by medical or nursing staff. Based on that, a percentage score of medical performance for each team is generated.

### Ethical Considerations

The local institutional review and ethics board judged the project as not representing medical or epidemiological research on human participants and as such adopted a simplified assessment protocol. The project was approved without any reservation (proposal 20240422-01). Students will be informed about the study and their participation is voluntary. Written informed consent is obtained in printed form from all participants, who are also provided with information on data processing for the analysis and the publication of results. Contact details are supplied for participants wishing to withdraw their consent to data processing. The decision to participate or not has no consequences on the students’ academic progress. Survey data from the questionnaires are collected anonymously using the EvaSys platform. Data are processed and stored in accordance with local data protection laws. A €30 (approximately US $33.40) book voucher will be handed to the participants upon completion of day 3.

### Statistical Analysis

Test quality criteria of vTPOT will be assessed as follows: For concurrent validity, Pearson correlations will be calculated. Interrater reliability of the vTPOT will be calculated using Cohen Kappa to quantify the agreement between the assessments of the independent reviewers. Internal consistency of the vTPOT will be assessed by using Cronbach α. A value exceeding 0.7 will be considered acceptable, and greater than 0.8 as good. Considering the results of objective team performance, subjective teamwork perception, medical performance scores, and descriptive statistics including mean and SD will be calculated and presented in the format of mean and SD. Pearson correlations will be calculated to capture relationships between the results of different measurement instruments. The Shapiro-Wilk test is used to check for normal distribution. In case of a violation of the normality assumption, nonparametric tests (Wilcoxon rank test for group differences and Spearman test for correlations) are used. The calculations and generation of figures will be performed using GraphPad Prism (Version 10.1.2; GraphPad Software Inc).

## Results

This study received ethics approval in April 2024. As of May 2024, the enrollment of participants is ongoing and currently (March 2025) has 28 enrolled interprofessional teams. Data collection and analysis will be complete in December 2025.

## Discussion

### Strengths and Limitations

This study leverages advanced VR technology to enhance interprofessional team training in medical education, allowing participants to engage in immersive, high-fidelity scenarios that closely mimic real-life emergency situations. A key strength lies in the use of an established VR-based training program (STEP-VR) that has been implemented in curricular teaching at several locations in Germany since 2020 and has already been evaluated in various teaching and examination settings. In terms of teamwork, the well-researched and validated measurement instruments of the TeamSTEPPS framework are used as a foundation, which is optimally aligned with teamwork training. The adaptation and validation are carried out in a rigorous selection process together with experts from all involved professions. Thus, the results will provide both objective and subjective insights into the improvement of teamwork skills. In addition, the study’s relatively large sample size and inclusion of participants from different cohorts within nursing and medical programs offer a comprehensive view of the potential impact of VR-based training on interprofessional teams.

Despite these strengths, there are several limitations to consider. First, the absence of a control group limits our ability to definitively attribute improvements in teamwork to the VR-based training intervention alone. The pre-post design allows for intragroup comparisons, but the lack of a parallel group experiencing conventional training or no training reduces the robustness of our findings. In particular, regarding the subjectively perceived quality of teamwork, an anticipated treatment effect of uncertain magnitude could arise due to the lack of a control group. To mitigate this, we reassemble the teams for each session. While an effect due to mere repetition is also conceivable for the objective measurements of teamwork quality, it is less likely, as most TeamSTEPPS concepts (eg, check-backs, the Two-Challenge Rule) are unlikely to be learned intuitively. Moreover, the intervention (the training video on successful teamwork) is significantly shorter than typical training sessions from the TeamSTEPPS framework and may not achieve a sufficient effect. However, with regard to future application, it seemed important to find a training concept that could be applied both in academic studies and within the tightly scheduled clinical routine. In addition, the study is conducted at a single institution, which may restrict the generalizability of the results to other educational contexts or healthcare systems. Finally, another limitation stems from the voluntary nature of participant enrollment. It is possible that those opting to participate may already have a positive attitude toward teamwork or technology, potentially introducing a selection bias.

### Challenges

One of the main challenges in this study is the adaptation of the TPOT and T-TPQ to VR environments. VR scenarios differ from traditional simulation in their representation of communication and interaction, as some elements (eg, natural communication with patients and their relatives) may be missing and others (eg, nonverbal cues and nuanced interpersonal behaviors) are often not as effectively captured in virtual environments. To address this, the vTPOT has been developed, but further validation will be necessary to confirm its reliability and validity in virtual settings. In addition, the need for evaluators to accurately observe and assess participant actions in a VR scenario poses a challenge. Ensuring a clear and comprehensive view of participant behaviors, both in real-time and via recordings, will be crucial for the consistent and objective application of the vTPOT. Finally, recruiting participants could prove challenging, as the curricula of the different study programs vary significantly and include shift work during practical placements.

### Future Implications

To address some of the study’s limitations, future research could incorporate a control group receiving traditional team training or no intervention, allowing for a more rigorous comparison of the effectiveness of VR-based training. A multicenter approach could also increase the generalizability of the findings by including participants from different educational institutions and health care settings. Moreover, future studies could explore the integration of more sophisticated haptic devices and natural language processing to enhance the realism of the VR environment and better capture the full range of teamwork behaviors. As VR technology continues to advance, it will be essential to continuously refine the tools used for teamwork assessment to ensure they remain aligned with the evolving capabilities of the technology.

## Data Availability

The datasets generated during and analyzed during this study will be available from the corresponding author on reasonable request.

## Appendix

Multimedia Appendix 1 Medical actions to be performed during the virtual reality scenarios.

## Abbreviations

HMD: head-mounted display
TPOT: Team Performance Observation Tool
T-TPQ: Teamwork Perceptions Questionnaire
VR: virtual reality
vTPOT: adapted version of the TPOT
