# Supplementary material for: Training and Assessing Teamwork in Interprofessional Virtual Reality–Based Simulation Using the TeamSTEPPS Framework: Protocol for Randomized Pre-Post Intervention Study
Source: JMIR Res Protoc. 2025 May 27;14:e68705. doi: 10.2196/68705 (PMC12152433; doi:10.2196/68705)
Supplement: Multimedia Appendix 1 [file resprot_v14i1e68705_app1.docx]

**Table S1.**

| **Medical Actions: Scenario 1 – EVB** |
| --- |
| Indication for gastroscopy |
| Successful hemodynamic stabilization (mean arterial pressure > 65 mmHg) |
| Collection of emergency laboratory tests (hemoglobin, coagulation, lactate) |
| Transfusion of packed red blood cells |
| Volume replacement with crystalloids |
| Administration of a prokinetic agent (IV erythromycin) |
| Administration of proton pump inhibitors in case of initially unclear bleeding source |
| Acute reduction of portal vein pressure (via vasoconstrictor) |
| Intravenous antibiotic therapy (covering gram-negative spectrum) |
| **Medical Actions: Scenario 2 – COPD** |
| Performance of blood gas analysis |
| Suctioning of purulent sputum |
| Administration of broad-spectrum antibiotic therapy |
| Performance of microbiological diagnostics including sputum sample |
| Performance of a 12-lead ECG for differential diagnosis of cardiac origin |
| Indication for non-invasive ventilation in hypercapnic failure |
| Ordering of a chest X-ray examination |
| Symptomatic relief of dyspnea with morphine administration |
| Administration of bronchodilator therapy with ß2-mimetics or anticholinergics |
| Administration of anti-inflammatory therapy with prednisolone |
| **Medical Actions: Scenario 3 – AF-UTI** |
| Administration of effective anticoagulant drugs |
| Successful pharmacological control of heart rate |
| Treatment of urinary retention by insertion of bladder catheter |
| Administration of appropriate empirical antibiotics |
| Cardioversion only after exclusion of thrombi |
| Performance of microbiological diagnostics, including urinary samples |
| Ultrasound examination of the urinary tract |
| Administration of fluids (after treatment of urinary obstruction) |
| Recording of a 12-lead ECG |
| Request for a urological consultation |

AF-UTI: atrial fibrillation due to complicated urinary tract infection, COPD: chronic obstructive pulmonary disease, ECG: electrocardiogram, EVB: esophageal variceal bleeding, IV: intravenous
